# Error-Based Observer of a Charge Couple Device Tracking Loop for Fast Steering Mirror

**DOI:** 10.3390/s17030479

**Published:** 2017-02-28

**Authors:** Tao Tang, Chao Deng, Tao Yang, Daijun Zhong, Ge Ren, Yongmei Huang, Chengyu Fu

**Affiliations:** 1 Key Laboratory of Optical Engineering, Chinese Academy of Science, Chengdu 610209, China; chaosir1991@gmail.com (C.D.); Yangtao@gmail.com (T.Y.); zqfirefly@126.com (D.Z.); renge@ioe.ac.cn (G.R.); huangym@ioe.ac.cn (Y.H.); cyfu@ioe.ac.cn (C.F.); 2 Institute of Optics and Electronics, Chinese Academy of Sciences, Chengdu 610209, China; 3 University of Chinese Academy of Sciences, Beijing 100039, China

**Keywords:** CCD error-based observer, fast steering mirror, image sensor, light of sight error, low-rate sample, time delay

## Abstract

The charge couple device (CCD) tracking loop of a fast steering mirror (FSM) is usually used to stabilize line of sight (LOS). High closed-loop bandwidth facilitates good performance. However, low-rate sample and time delay of the CCD greatly limit the high control bandwidth. This paper proposes an error-based observer (EBO) to improve the low-frequency performance of the CCD tracking system. The basic idea is by combining LOS error from the CCD and the controller output to produce the high-gain observer, forwarding into the originally closed-loop control system. This proposed EBO can improve the system both in target tracking and disturbance suppression due to LOS error from the CCD’s sensing of the two signals. From a practical engineering view, the closed-loop stability and robustness of the EBO system are investigated on the condition of gain margin and phase margin of the open-loop transfer function. Two simulations of CCD experiments are provided to verify the benefits of the proposed algorithm.

## 1. Introduction

A charge couple device (CCD)-based fast steering mirror (FSM) control system is widely used in adaptive optics, free space communication, and line of sight stabilization [1,2,3]. The CCD image sensor is crucial both in observation and orientation, and it is generally applied to detect the centroid of a target source to provide the line of sight (LOS) error to implement the closed-loop control system. In long-distance image detection such as deep space communication, the CCD usually needs a relatively large amount of integral time for a high definition image. Low-rate frame and time delay are unavoidable in the CCD tracking loop. The major limitation to a CCD-based tracking loop is the time delay [4,5], which can significantly reduce the closed-loop performance. Even though there are many articles concerning the CCD-based control problem, the work [6,7] usually concentrates on upgrading hardware and software architectures. The predictive control [8] is considered to be an effective method for compensating time delay. However, the predictive control is only efficient in low measurement noise conditions because a predictor cannot predict white noise. An acceleration feedback loop [1,9] was introduced to enhance the tracking and pointing performance. An acceleration loop rebuilds the control plant into the integral type in the FSM control system, but it brings an additional sensor and there is quadratic differential effect in the open-loop transfer function in the low-frequency range. In fact, improving the performance at low frequencies is more important than high-control bandwidth. A feedforward controller [10] combining an LOS error with an angular sensor is proposed to compensate for errors related to time delay in the CCD tracking loop of a fast steering mirror. An additional position sensor is required to recover the target trajectory for implementing feedforward control. A simple PID-I (proportional-integral-differential) controller [11] is proposed in the CCD loop of the FSM control system, which is to add an integrator into the traditional PID controller. However, two integrators in the control system easily lead to integral saturation. This paper proposes an error-based observer (EBO) to improve the low-frequency performance of the CCD tracking loop. The EBO combines the LOS error from the CCD and the controller output to produce a high-gain observer, and feedforwards into the originally closed-loop control system. The proposed EBO can benefit the control system both in target tracking and disturbance suppression because of the LOS error from the CCD’s sensing of the two signals. From a practical engineering standpoint, the closed-loop stability and robustness of the EBO system are investigated on the condition of gain margin and phase margin of the open-loop transfer function. In this control mode, there is no extra sensor except CCD to implement the EBO.

Section 2 presents a detailed introduction to the EBO, mainly describing the mathematical model of the CCD-based tracking system. Section 3 focuses on parameters design, to be specific in terms of controller and low-pass filter. Section 4 discusses and analyzes system performance. Section 5 sets up simulations and experiments to testify the theorems in Section 4. Concluding remarks are presented in Section 6.

## 2. The Model of CCD-Based FSM System

**Figure 1:**
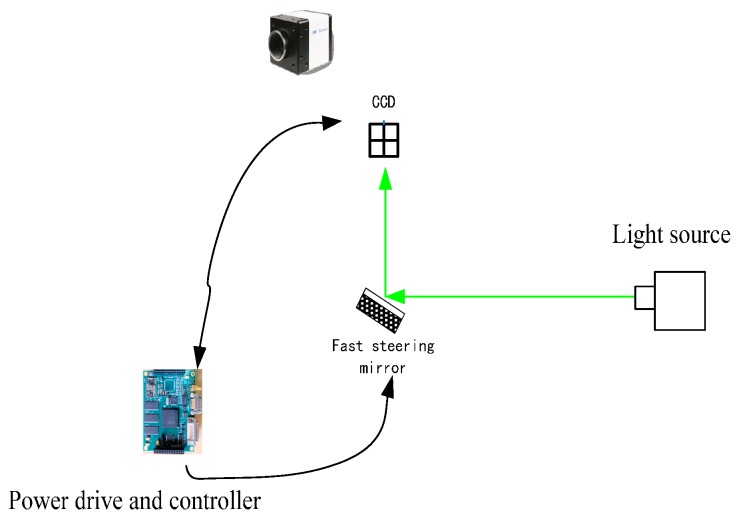
Configuration of the charge couple device (CCD) control system.

The configuration of a CCD-based tracking system is illustrated in Figure 1. The CCD as a tracker provides the LOS error for controlling the FSM. The light source, through the FSM and disturbance mirror to the CCD, is used to simulate the target. The controller hardware is used to implement the control algorithm. The power driver actuates the FSM to achieve the tracking control. 

The CCD characteristics in this system are shown in Table 1.

**Figure 2:**
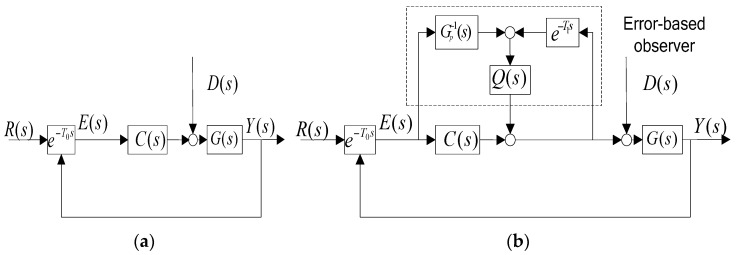
The control mode. (**a**) The traditional control mode; (**b**) the error-based observer (EBO) control mode.

The maximum focus length is 1000 mm, so the instantaneous visual field is about 5.5 μ∙rad. The control structure of Figure 1 is shown in Figure 2, which includes the CCD tracking loop and the observer based on the LOS error. Q(s) is the low-pass filter. G(s) is the control plant. C(s) is the position controller. The time delay $e^{-T_{0}s}$ characterizes the CCD in the control system although it may be rough. The function $e^{-T_{1}s}$ depicts the total time delay. R(s) represents the target trajectory but not the availability. E(s) is the LOS error from the CCD. D(s) is the disturbance, which might not be measured by the sensors. Y(s) stands for the output. $G_{p}^{-1}(s)$ describes the inverse of the control plant G(s). Obviously, it is impossible to have a perfectly accurate model of G(s) in the high-frequency region, evenly in the middle-frequency region.

The block diagram in Figure 2 applies to regulation and tracking control. Different from reducing closed-loop error E(s) in the presence of the disturbance, we aim at maintaining tracking error small without considering disturbance. In a CCD-based tracking loop, the time delay greatly limits the closed-loop bandwidth. In addition, the high-velocity position loop can build G(s) into several hundred Hz bandwidth [6,11]. Thus, G(s)≈1 is reasonable below the closed-loop bandwidth.

The controller $C(s)=\pi /4T_{0}s$ [12] can stabilize plant G(s) to obtain a phase margin more than 35° and magnitude margin more than 6 dB. Defining $w_{c}$ as the crossover frequency and $w_{g}$ as the gain frequency of the open-loop transfer function $G_{open}(s)=C(s)G(s)$, we can easily obtain $w_{c}=\pi /4T_{0}$ and $w_{g}=\pi /2T_{0}$. The open-loop sensitivity transfer function [13] illustrated in Figure 2b is given as follows:

(1) $$S_{R}=\frac{E(s)}{R(s)}=\frac{1-e^{-T_{1}s}Q}{1+CGe^{-T_{0}s}+(GG_{p}^{-1}e^{-T_{0}s}-e^{-T_{1}s})Q}=(1-e^{-T_{1}s}Q)S_{R}^{\prime }$$

(2) $$S_{D}=\frac{E(s)}{D(s)}=\frac{(1-e^{-T_{1}s}Q)G}{1+CGe^{-T_{0}s}+(GG_{p}^{-1}e^{-T_{0}s}-e^{-T_{1}s})Q}=(1-e^{-T_{1}s}Q)S_{D}^{\prime }$$

Here, $S_{R}^{\prime }$ and $S_{D}^{\prime }$ are the original error and disturbance attenuation functions, respectively, in Figure 2a. Obviously, minimizing $1-e^{-T_{1}s}Q(s)$ is the objective. It is clear that the term $1-e^{-T_{1}s}Q(s)$ cannot be expected to be zero, but it could be designed close to zero in a low-frequency domain, wherein the main frequencies of the tracking target R(s) are located. Therefore, Q(s) is expressed as a low-pass filter because of $1-e^{-T_{1}s}Q(s)$ characterizing high-pass filter for improving low-frequency performance. Furthermore, it is practically impossible to have a perfectly accurate model of G(s) in the high-frequency region, and thus necessary to incorporate a low pass filter in Q(s) to make the influence of $(GG_{p}^{-1}e^{-T_{0}s}-e^{-T_{1}s})Q$ small. From the control stability’s standpoint, Q(s) should have a bandwidth as low as possible. However, the situation is opposite against the improvement of $1-e^{-T_{1}s}Q(s)$. Clearly, the improvement of the proposed control mode in Figure 4 is not subject to the control plant, dependent only on the low-pass filter and time-delay function.

## 3. Parameters Design

The equivalent controller in Figure 2b is given in Equation (3).

(3) $$C_{eq}(s)=\frac{G_{p}^{-1}Q+C}{1-e^{-T_{1}s}Q}$$

$C_{eq}(s)$ is always a stabilizing controller for the plant, because this is the output of the simplest form of Youla parameterization as long as Q(s) is stable and rational [14].

(4) $$G_{open}^{\prime }=\frac{C^{-1}G_{p}^{-1}Q+1}{1-e^{-T_{1}s}Q}CGe^{-T_{0}s}$$

G(s) is similar to one, resulting in making $C^{-1}G_{p}^{-1}Q\approx C^{-1}Q=ksQ$ where $k=4T_{0}/\pi$. Q(s) is expressed as a simplest low-pass filter as follows:

(5) $$Q=\frac{1}{\tau s+1}$$

In fact, for the equivalent controller not to affect the closed-loop stability margin, there are two necessary constraints: (1) $\arg[A(jw_{c})]\geq 0$; (2) $-20\log[A(jw_{g})]-20\log\left|G_{open}(jw_{g})\right|\geq 6\;\mathrm{dB}$, where A(s) is defined as follows:

(6) $$A(s)=\frac{C^{-1}G_{p}^{-1}Q+1}{1-e^{-T_{1}s}Q}\approx \frac{ks+(\tau s+1)}{(\tau s+1)-e^{-T_{1}s}}$$

For analyzing the characteristic of A(s), let $e^{-jT_{1}w}=\cos(T_{1}w)-j\sin(T_{1}w)$. Thus, we have

(7) $$A(jw)=\frac{j(kw+\tau w)+1}{1-\cos(T_{1}w)+j[\tau w+\sin(T_{1}w)]}$$

Due to the existence of sine and cosine function, A(jw) fluctuates both in phase function and magnitude function at every period of the sine function. If we define M(w) as phase function of A(jw), then we have

(8) $$M(w)=ac\tan(kw+\tau w)-ac\tan\frac{\tau w+\sin(T_{1}w)}{1-\cos(T_{1}w)}$$

if $T_{1}=0$ then M(w)=actan(kw+τw)−0.5π<0; therefore, τ needs to be large enough to make the phase loss of M(w) small at the crossover frequency of $w_{c}$. However, large τ results in sacrificing the improvement of the closed-loop performance. Considering the derivative of M(w) below,

(9) $$M^{\prime }(w)=\frac{\tau +k}{1+{\left(\tau +k\right)}^{2}w^{2}}-\frac{\left(\tau -T_{1})[1-\cos(T_{1}w)]-\tau T_{1}w\sin(T_{1}w\right)}{{\left[1-\cos(T_{1}w)\right]}^{2}+{\left[\tau w+\sin(T_{1}w)\right]}^{2}}$$

$M^{\prime }(w)>0$ is obvious if $\tau -T_{1}\leq 0$ when $T_{1}w$ increases from 0 to π. $M^{\prime }(w)>0$ is still confirmed when $\tau -T_{1}>0$.

**Proof.**  We consider two cases below:

(1) $T_{1}w\in (0,\frac{1}{2}\pi ]$

If ϕ(w)<0, $M^{\prime }(w)>0$ is obvious, where $\phi (w)=(\tau -T_{1})[1-\cos(T_{1}w)]-\tau T_{1}w\sin(T_{1}w)$. We easily obtain ϕ(0)=0 and $\phi^{\prime }(w)=T_{1}^{2}[\sin(T_{1}w)+\tau w\cos(T_{1}w)]<0$. Therefore, $\phi (T_{1}w)<0$ such that $M^{\prime }(w)>0$.

(2) $T_{1}w\in [\frac{1}{2}\pi ,\pi ]$

Because of $0.5\leq \frac{1}{1-\cos(T_{1}w)}\leq 1$ and $0<\cot\frac{T_{1}w}{2}<1$, so

(10) $$\begin{array}{l} M(w)=ac\tan(kw+\tau w)-ac\tan[\frac{\tau w}{1-\cos(T_{1}w)}+\cot\frac{T_{1}w}{2}]\\ >ac\tan(kw+\tau w)-ac\tan[\tau w+1] \end{array}$$

From the above analysis, M(w) increases monotonously as $T_{1}w$ changes from 0 to π. Note that $M_{w\to 0}=-0.5\pi$ and $M_{w\to \infty }=0$ are obvious. $M(w_{c})=0$ is expected when $T_{1}w_{c}=0.5\pi$ such that $T_{1}=0.5\pi k$ is obtained. ☐

The magnitude function is depicted as follows:

(11) $$20\log\left|A(jw)\right|=10\log[\frac{{\left(wk+w\tau \right)}^{2}+1}{{\left[1-\cos(T_{1}w)\right]}^{2}+{\left[\tau w+\sin(T_{1}w)\right]}^{2}}]$$

It is obvious $20\log\left|A(j\infty )\right|=20\log(\frac{k}{\tau }+1)$, so τ≥k is expected for reducing the loss of magnitude margin. At the gain frequency of $w_{g}$, $\left|A(jw_{g})\right|$ is given in in Equation (12):

(12) $$20\log\left|A(jw_{g})\right|=10\log[1+\frac{4\tau w_{g}+1}{4+\tau^{2}w_{g}^{2}}]$$

We can easily obtain the maximum $\max\;20\log{\left|A(jw_{g})\right|}_{\tau w_{g}=\frac{\sqrt{65}-1}{4}}\approx 6.4$. Because $20\log\left|G_{open}(jw_{g})\right|=-6.5$, it is almost impossible to let $20\log\left|G_{open}^{\prime }(w_{g})\right|\geq 6$ as long as τ is limited. Therefore, for a phase margin more than 35° and a magnitude margin more than 6 dB [15], with this proposed EBO, the original controller C(s) is replaced with 0.8C(s) coupled with $\tau =3.0T_{0}$ and $T_{1}=1.66T_{0}$.

## 4. Performance Analysis

With the proposed EBO, the closed-loop performance is dependent on the characteristics of $1-e^{-T_{1}s}Q(s)$. The effect of $1-e^{-T_{1}s}Q(s)$ in the closed-loop system is analyzed in terms of attenuation bandwidth and amplification value. We substitute $e^{-jT_{1}w}=\cos(T_{1}w)-j\sin(T_{1}w)$ into $1-e^{-T_{1}s}Q(s)$, yielding

(13) $${\left|1-e^{-jT_{1}w}Q(jw)\right|}^{2}=1+\frac{1+2\tau w\sin(T_{1}w)-2\cos(T_{1}w)}{{\left(\tau w\right)}^{2}+1}).$$

${\left|1-e^{-jT_{1}w}Q(jw)\right|}^{2}=1$ means $1+2\tau w\sin(T_{1}w)=2\cos(T_{1}w)$, which implies the minmum w meets $0.16\pi <T_{1}w<0.25\pi$. Substituting $\tau =3.0T_{0}$ and $T_{1}=1.66T_{0}$ into the above equation coupled with $w_{c}T_{0}=0.25\pi$, the attenuation bandwidth is about $0.5w_{c}$.

The below analysis is about amplification of $1-e^{-T_{1}s}Q(s)$. Rewriting Equation (13) into Equation (14),

(14) $${\left|1-e^{-jT_{1}w}Q(jw)\right|}^{2}=1+\frac{1+2\sqrt{{\left(\tau w\right)}^{2}+1}\sin(T_{1}w-\beta )}{{\left(\tau w\right)}^{2}+1}\leq 1+\frac{1+2\sqrt{{\left(\tau w\right)}^{2}+1}}{{\left(\tau w\right)}^{2}+1}$$

where $\beta =\arccos\frac{\tau w}{\sqrt{{\left(\tau w\right)}^{2}+1}}$ is between 0 and 0.5π is a strictly decreasing function as the frequency increases.

Considering $\tau w=2.25T_{1}w$, $T_{1}w-\beta =0.5\pi$ is possible. Thus, Equation (14) can obtain the maximum value. Because $\tau w=2.25T_{1}w=3.53$ when $T_{1}w=0.5\pi$, $\sqrt{{\left(\tau w\right)}^{2}+1}$ approximates to τw. Reasonably, the maximum value $\left|1-e^{-jT_{1}w}Q(jw)\right|$ occurs at $T_{1}w=0.5\pi$, which is approximated to 1.36. In other words, its maximum amplification is about 3 dB compared with the originally closed-loop system at the same frequencies.

**Figure 3:**
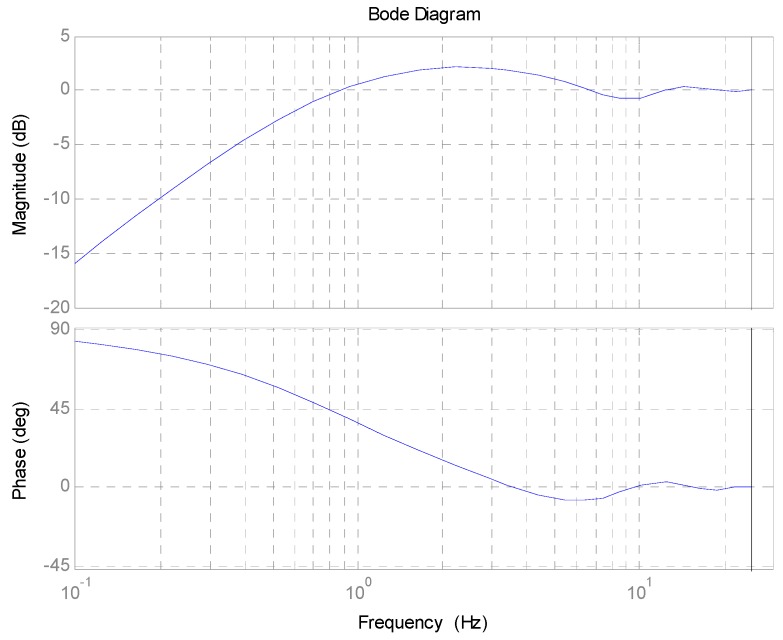
Bode response of $1-e^{-T_{1}s}Q(s)$.

Based on aforementioned considerations and design, the Bode response of $1-e^{-T_{1}s}Q(s)$ is shown in Figure 3. The cutoff frequency is about 0.9 Hz, which implies that it can improve the closed-loop performance below the cutoff frequency. A large attenuation of more than −10 dB is achieved below the frequency of 0.1 Hz. However, the magnitude response in the middle-frequency range from 0.9 Hz to 5.5 Hz is magnified due to the amplification by exponential function and low-pass filter. This phenomenon absolutely respects Bode’s integral theorem.

**Figure 4:**
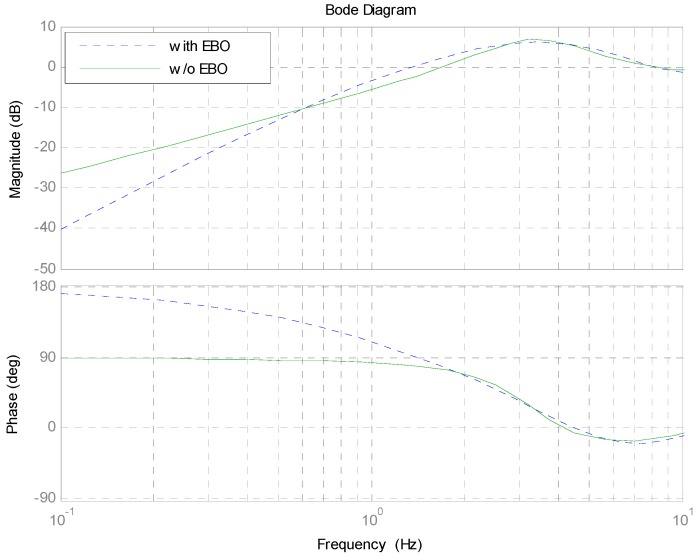
Bode response of error attenuation.

The Bode response of error attenuation depicted in Equation (1) is shown in Figure 4. The control system with the EBO improves below the frequency of 0.6 Hz, lower than the cutoff frequency of $1-e^{-T_{1}s}Q(s)$. The major cause is that the influence of $(GG_{p}^{-1}e^{-T_{0}s}-e^{-T_{1}s})Q$ cannot be cut into zero, resulting in deteriorating middle-frequency performance of the closed-loop system. It is impossible to obtain a precision model of the G above the low-frequency region, such that $GG_{p}^{-1}e^{-T_{0}s}-e^{-T_{1}s}$ is not approximated to zero. Therefore, choosing a low-pass filter can contribute significantly to reducing this enlargement, but the improvement of the $1-e^{-T_{1}s}Q(s)$ must degrade.

## 5. Experimental Setup

**Figure 5:**
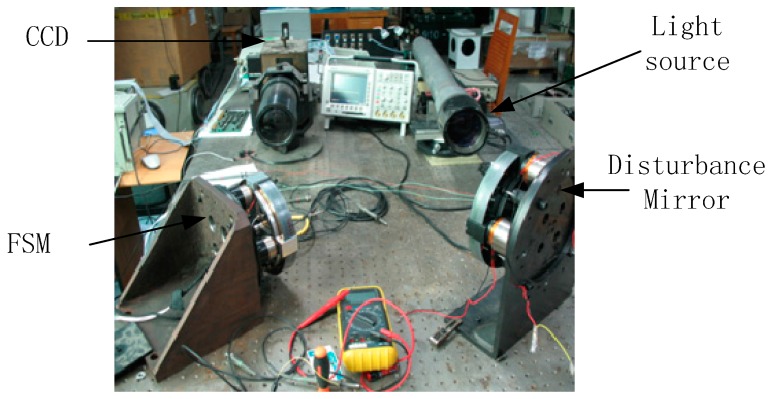
Experimental setup of the CCD control system.

The experimental setup of the CCD-based tracking system is illustrated in Figure 5. The disturbance mirror is also an FSM, moving in both X-Y directions to simulate the vibration of the light source. The CCD as a tracker is placed in the focal plane of an imaging lens to detect the centroid of the target source to provide the LOS error. The CCD updates in 50 Hz, and the delay parameter $T_{0}$ is about 0.06 s. From the ahead analysis and design, we have τ=0.18 and $T_{1}=1.0$.

**Figure 6:**
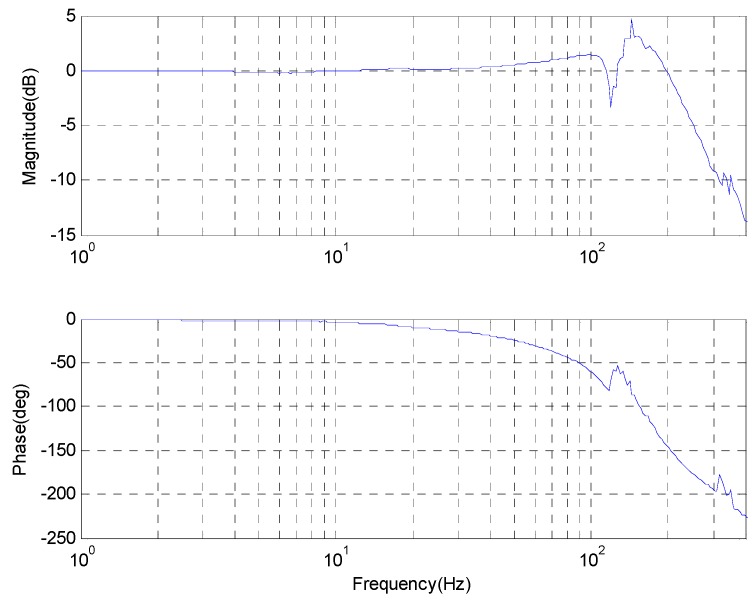
The Bode response of the control plant.

The Bode response of the control plant is given in Figure 6, and its bandwidth is more than 150 Hz. The maximum magnitude relative to 0 dB is less than 3 dB below the frequency of 30 Hz, while the phase loss relative to 0° is less than 6°, shown in Figure 5. Thus, the closed-loop transfer function approximates to unit below the low frequency of 30 Hz. 

**Figure 7:**
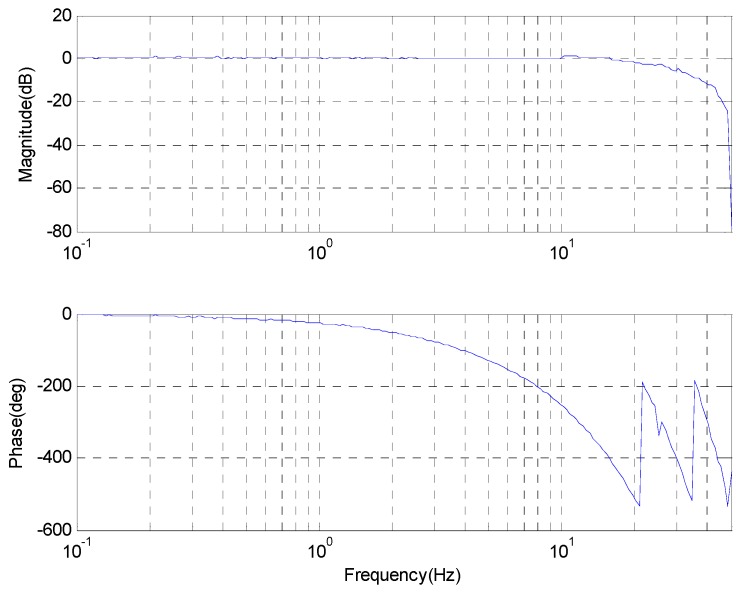
The CCD open-loop Bode response.

Due to the large amount of time delay in the CCD tracking loop, the CCD open-loop response in Figure 7, in which a pure delay appears to decrease in phase sharply after the frequency of 1 Hz, while the magnitude remains strong until the frequency reaches 16 Hz. Therefore, a simple integral controller is beneficial to achieve high closed-loop performance.

**Figure 8:**
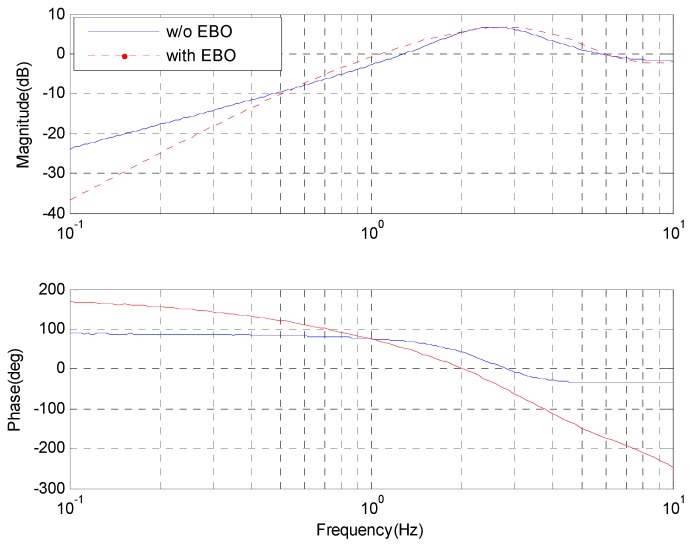
The experimental error attenuation.

The error attenuation bandwidth (about 1.2 Hz) with the EBO controller is lower than that (about 1.5 Hz) with the only integral controller in Figure 8. In the frequency range of about 0.5~1 Hz, the closed-loop performance without the EBO is a little better, which looks very much like simulations shown in Figure 4. Furthermore, it shows that the performance improves about 10 dB below the frequency of 10 Hz. Fortunately, the experimental results are in accordance with the theoretical analysis.

## 6. Conclusions

A high-gain controller based on LOS error for a CCD tracking loop is proposed to improve the low-frequency performance of the FSM closed-loop system. In this paper, we focus on the implementation of the EBO, the optimization of the control parameters, and the analysis of the close-loop stability from the viewpoint of its practical implementation. The conditions for implementing the EBO into the CCD-based tracking loop for FSM were presented mainly in terms of the closed-loop stability and error attenuation. Although the control bandwidth is not obviously improved with this proposed controller, the low-frequency error attenuation is enhanced. Different from the classical feedforward controller [10], the improvement of this proposed controller is not subject to the control plant. Experiments verify this technique effectively to enhance closed-loop performance in comparison with the classical PID control mode. 

## Figures and Tables

**Table 1:** CCD parameters.

Frame frequency	50 Hz
Pixels	640 × 512
Pixel size	5.5 μm
Focus length	500 mm–1000 mm
